# Malaria prevalence in asymptomatic and symptomatic children in Kiwangwa, Bagamoyo district, Tanzania

**DOI:** 10.1186/s12936-017-1870-4

**Published:** 2017-05-25

**Authors:** Deborah Sumari, Felista Mwingira, Majige Selemani, Joseph Mugasa, Kefas Mugittu, Paul Gwakisa

**Affiliations:** 1 Intervention and Clinical Trials Department, Ifakara Health Institute, Bagamoyo, Tanzania; 2 School of Life Sciences and Bioengineering, The Nelson Mandela African Institution for Science and Technology, Arusha, Tanzania; 3 Biological Sciences Department, Dar es Salaam University College of Education, P. O. Box 2329, Dar es Salaam, Tanzania; 4 Department of Statistics, University of Dar es Salaam, P. O. Box 35047, Dar es Salaam, Tanzania; 5 National Institute for Medical Research, Amani Medical Research Centre, P. O. Box 81, Muheza, Tanga, Tanzania; 6 Muvek Laboratories, P. O. Box 105270, Dar es Salaam, Tanzania; 7 Genome Sciences Centre and Department of Microbiology, Parasitology and Immunology, College of Veterinary and Medical Sciences, Sokoine University of Agriculture, P. O. Box 3019, Morogoro, Tanzania

**Keywords:** Symptomatic malaria, Asymptomatic malaria, *Plasmodium falciparum*, Tanzania, Quantitative PCR

## Abstract

**Background:**

Malaria prevalence continues to decline across sub-Saharan Africa as a result of various intervention strategies. However, the diseases still poses a public health concern in the region. While symptomatic malaria is recognized and treated, asymptomatic infections become increasingly important for interrupting transmission. A cross-sectional survey was conducted to assess malaria prevalence in symptomatic and asymptomatic children in Kiwangwa ward in Bagamoyo District in Tanzania.

**Methods:**

Four hundred school-aged children in Kiwanga ward were recruited in the study; 200 from Kiwangwa dispensary and 200 from nearby schools. Primary health parameters were examined and blood samples collected and examined for *Plasmodium falciparum* prevalence using rapid diagnostic test (RDT), light microscopy (LM) and reverse transcription quantitative PCR (RT-qPCR) targeting transcripts of A-type 18s rRNA of *P. falciparum*. Gametocytes were detected by LM and RT-qPCR targeting transcripts of gametocyte specific marker, *Pfs25*.

**Results:**

Overall *P. falciparum* prevalence was 73.3, 40.8 and 36.3% by RT-qPCR, RDT and LM in the study area, respectively (P < 0.001). As expected symptomatic children had a significantly higher prevalence of 89, 67.5 and 64.5% by qPCR, RDT and LM, compared to 57.5, 14 and 8% in the asymptomatic group, respectively. However, gametocyte prevalence in asymptomatic individuals was higher by both LM (2%) and qPCR (14%) than in symptomatic individuals LM (0.5%) and qPCR (3%).

**Conclusions:**

A substantial difference in prevalence of symptomatic and asymptomatic infections observed in Kiwangwa ward underpins the use of molecular tools in malaria surveillance aiming at estimating prevalence and transmission. Notably, the higher gametocytaemia observed in asymptomatic children indicates the reservoir infections and points to the need for detection and treatment of both asymptomatic and symptomatic malaria.

## Background

Infection with *Plasmodium falciparum* can result into asymptomatic carriage, uncomplicated or severe malaria [1]. In endemic areas, many people experience asymptomatic *Plasmodium* infections particularly older children and adults where the different manifestation of malaria cases is a function of immunity with respect to age and exposure to the parasite [2]. In high malaria transmission settings, symptomatic malaria is often in children below 5 years of age as they have little exposure hence weak/low immunity to the parasites [3]. On the other hand, asymptomatic infections and gametocytes usually occur in older children and at submicroscopic densities, challenging their diagnosis in the population [4]. Asymptomatic infections are known to be prevalent in low endemic areas and are generally untreated. Prolonged and persistent infections are known to encourage gametocytogenesis, resulting in a significant source of malaria transmission [5] where more sensitive molecular techniques allow better characterization and quantification of gametocyte patterns in the population.

Asymptomatic infections in many endemic areas have become highly prevalent [6, 7] which brings a new challenge for malaria prevention and control strategies in Sub-Saharan Africa. The prevalence of asymptomatic parasitaemia in a given population is inversely related to the population’s susceptibility to clinical disease [8]. Therefore, monitoring changes in prevalence rates is useful for measuring outcomes of antimalarial interventions or for informing control strategies.

The prevalence of symptomatic and asymptomatic infections in malaria endemic areas has been well documented [9] but, there is insufficient information on the prevalence of *P. falciparum* infections and gametocytes carriage both in asymptomatic and symptomatic individuals following reduction of malaria burden. It is well known that asymptomatic infections go undetected and untreated with little or no clinical manifestation [10]. However, asymptomatic individuals remain the major reservoir of infection and are largely responsible for sustaining malaria parasite population between transmission seasons [11].

Malaria surveys that use only LM and RDT as diagnostic tools, usually fail to detect low‐level parasitaemia [12] that is common among asymptomatic people. Introduction of molecular assays for parasite and gametocyte detection [13, 14] to epidemiological surveys have improved the overall detection of low levels and asymptomatic parasitaemia, leading to better estimation of malaria burden and their potential role in the transmission of malaria in human populations. A strong focus on malaria elimination needs to interrupt transmission by using proper identification and treatment of all carriers both symptomatic and asymptomatic. Therefore, this study aimed at establishing and comparing parasite prevalence of asymptomatic and symptomatic individuals and the extent of parasite reservoir in primary school-aged children in a selected area in Bagamoyo, Tanzania.

## Methods

### Study design

This was a cross-sectional study conducted in Kiwangwa ward in Bagamoyo. This is a study was a subset of a main study which was conducted in Bagamoyo district. The study was conducted during high malaria transmission seasons. A total of 400 primary school children aged 6–14 years in Kiwangwa ward of Bagamoyo district were recruited during high malaria transmission season between June and August while other groups were recruited during the second high season from October to December 2014. Two hundred blood samples were collected from symptomatic children with uncomplicated malaria seeking health care at Kiwangwa health facility. At health center, all children aged 6–14 years with malaria and consented to be recruited to the study were included, whereas the remaining 200 blood samples were obtained from asymptomatic school children using a simple purposively randomly selection from Msinune (n = 40), Mwavi (n = 43), Fukayosi (n = 37) and Kidomole (n = 80) primary schools. The number of participants from each school was based on recruitment of only consented participants and parent(s)/guardians(s). The study site was selected based on a previous survey on malaria prevalence conducted in 2011/2012 in the sentinel district of Bagamoyo villages. The survey identified Kiwangwa as a high malaria endemic area in Bagamoyo district (Unpublished data, IHI) and logistically it supported sample collection and transportation of molecular samples to the Bagamoyo laboratory which is located 30–45 min away from the study sites. A semi-structured interview guide combining both closed and open-ended questions was used to collect demographic data of the participants. The sampling of symptomatic and asymptomatic children was done at the health facility and at the schools, respectively.

The sample size was calculated using a standard formula for prevalence studies [15] as follows:

$$n = \frac{{Z^{2} P\,(1 - P)}}{{d^{2} }}$$

where *n* is sample size, *Z* is a *Z* statistic value of 1.96 at confidence level of 95%. *P* is considered prevalence at 10% [16] at 95% confidence interval and *d* is a 5% relative precision. To account for dropout from school during the study, 20% of the calculated sample size was added to account for missing samples. Therefore, a design effect of two to account for clustering within health facility and nearby schools was used and the targeted sample size of 400 children was obtained to assess malaria prevalence in symptomatic and asymptomatic children in the study area.

Both symptomatic and asymptomatic malaria cases were referred and offered appropriate treatment according to malaria treatment guidelines [17, 18]. Exclusion criteria from the study were severe malaria and other chronic illness, age of less than 6 years or more than 14 years and lack of informed consent. Laboratory screening and other routine procedures were conducted according to standards of care.

## Ethical consideration

This study received ethical approval from Institutional Review Board No. IHI/IRB/No: 34-2013 of Ifakara Health Institute and Medical Research Coordinating Committee of the National Institute for Medical Research No. NIMR/HQ/R.8a/Vol.IX/1705. Regional, district and community authorities in the study area were contacted and granted a written approval of the study. Prior to participation, a written consent of each respondent was obtained based on Informed Consent Forms (ICFs) designed for the study. Additionally, the confidentiality of information obtained from study participants was assured by using unique identifiers. Each parent or guardian was given a form that explained risks, benefits and confidentiality that would have been taken. In case a parent or guardian was illiterate, school teachers and/or heath workers provided assistance to acquaint such parent/guardian with the study. Children were only included in the study after their parents and/or guardians, consented, signed and returned the ICFs to the study team through schools under study.

### Malaria screening and blood sampling procedures

#### RDT and LM

Three millilitres of venous blood was collected by a syringe from each participant for rapid diagnostic test (RDT), LM and RT-qPCR. Fifty microlitre of the sampled blood was spotted onto Whatman^®^ 3MM filter paper as dried blood spots (DBS) direct from the syringe before putting the rest of the blood sample into heparin vacutainer tubes. RDT (ICT Malaria Dual Cassette Test; ICT Diagnostics, Cape Town, South Africa) was done on the spot in the field and at the dispensary for quick malaria screening as described elsewhere [19]. A total volume of 6 and 3 µL of blood were used for preparation of thick and thin blood smears, respectively. The remainder blood was immediately stored in heparin vacutainer tubes which was used for other objectives of the study. Microscopy samples were prepared and examined on site, whereas quantification and detection of parasites stages were conducted in the laboratory which is 30–45 min away from the field [20]. As a quality control measure two microscopists examined the slides independently while a third microscopist reexamined any slides with discordance. Samples were considered negative if no parasites were detected in 100 high-power fields of Giemsa-stained thick blood smears. Both, asexual and sexual stages of the parasites were assessed in thick smears by comparing ratio of infected red blood cells to uninfected ones. Thin smears were examined for parasite speciation and quantification. Gametocytes and asexual stage parasites were counted against 500 and 200 white blood cells (WBCs), respectively, and densities (parasite per microlitre) were estimated using a factor of 8000 leukocytes/µL.

### Molecular investigation

Fifty microlitre of whole blood was collected and spotted on the filter papers and air dried but not on direct sunlight for molecular analysis. The dried filter papers were stored into different plastic zipped bags labelled with unique identification number, study number and date together with desiccants. Then each dried blood spot (DBS) was cut into 10–12 small pieces and transferred into microcentrifuge tubes containing 250 µL TRizol^®^reagent (Invitrogen, Carlsbad, CA, USA) and stored at −80 °C until RNA extraction.

### RNA extraction

RNA was extracted from all blood samples collected on 3MM filter papers stored in TRIzol reagent regardless their RDT status. The filter papers were transferred from TRIzol into 606 µL RLT analysis buffer mixed with β-mercaptoethanol (Qiagen RNeasy^®^ plus mini kit) and incubated for 15 min at 30 °C on a shaker at 1400 revolution per minute (rpm). After incubation, the cocktail was centrifuged for 30 s at 14,000 rpm and the aqueous phase was transferred to a gDNA eliminator column, following manufacturer’s instructions with minor modifications. Lastly RNA was eluted using 50 µL RNase-free sterile water and stored at −80 °C.

### Molecular detection of *P. falciparum* infections

The *P. falciparum* RNA based quantitative nucleic acid sequence reverse transcription polymerase chain reaction (RT-qPCR) assay for targeting the A-type 18S rRNA of the parasite was performed to determine parasite prevalence [21]. All samples identified positive by A-type 18S rRNA RT-qPCR assay were further analysed for gametocytes prevalence in the same volume. Primers and probes sequences are listed in Table 1. Gametocytes specific gene *Pfs*25 mRNA transcripts were detected in extracted materials using quantitative nucleic acid sequence reverse transcription polymerase chain reaction (RT-qPCR). *Pfs*25 is expressed only in *P. falciparum* in late stage gametocytes which shows limited polymorphism and is currently used to quantify gametocytes from field isolates [22]. *Pfs*25 transcripts were reverse transcribed and the resulting cDNA was amplified by RT-qPCR whereas the assay was performed on extracted RNA samples from asymptomatic individuals after complete gDNA removal. Quantification of *P. falciparum* parasites and gametocytes was calculated in copy numbers obtained from each individual sample per µL of blood using standard curves of known parasite concentrations.

**Table 1:** Primers and probes sequences

Gene	Primers	5′ → 3′	Probe 5′ → 3′	Labelled
A-18s	FW	TCCGATAACGAACGAGATCTTAAC	TAGCGGCGAGTACACTATA	FAM-BHQ1 MGB
	RV	ATTATAGTTACCTATGTTCAATTTCA		
Pfs 25	FW	GAAATCCCGTTTCATACGCTTG	TGTAAGAATGTAACTTGTGGTAACGGT	HEX-BHQ1
	RV	TGCAGTTTTAACAGGATTGCTTGT		

### Data management and analysis

Data collected using questionnaires were entered into Microsoft^®^ excel file and transferred to STATA version 11 for data cleaning and analysis. The main outcome variable was malaria prevalence for asymptomatic and symptomatic children obtained using LM, RDT and qPCR. Comparison of these outcomes between the school children and diagnostic test was performed. Other variables were age and sex of children. The two sample test for proportional was used to test for differences of malaria prevalence between symptomatic and asymptomatic children. Sensitivity and specificity analysis on RDT and LM in comparison with qPCR a reference method was conducted based on the parasite density of the children in two groups.

## Results

### Characteristics of study population

The characteristics of study participants recruited from Kiwangwa ward health facility as well as from schools are shown in Table 2. There were more females than males recruited from the schools (P = 0.05) while there was no significant difference between number of boys and girls recruited from the health facility. The proportion of febrile children from health facility was significantly higher compared to that from schools (P = 0.001). Additionally, the mean body weight was higher in school population compared to health facility, although the difference was not significant despite the fact that they were of the same age distribution (Table 2).

**Table 2:** Characteristics of the study population

Characteristics	Asymptomatic	Symptomatic	P value
	n	n	
Sex			
Male	72 (36%)	91(45.5%)	0.05
Female	128 (64%)	109 (54.5%)	
Temperature			
<37.5 °C	188 (94%)	66 (33%)	<0.001
≥37.5 °C	12 (6%)	134 (67%)	
Mean weight (kg: Min–Max)	34 (17.6–62)	20.7 (8.2–48)	
Mean age (years: Min–Max**)**	11 (6–14)	9 (6–13)	

When n = number of participants

### Malaria prevalence by LM, RDT and qPCR methods

**Fig. 1:**
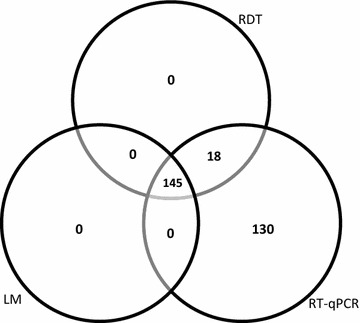
Venn diagram showing concordance in diagnostic tools for all malaria positive samples, n = 250

Overall, all three diagnostic methods; LM, RDT and qPCR detected significantly high malaria infections in symptomatic children from health facility (64.5, 67.5, 89%) compared to asymptomatic children from schools (8, 14 and 57.5%), respectively (Table 3). The average corresponding parasitemia density in symptomatic children and asymptomatic children was 27,100 parasites/µL and 82 parasites/µL, respectively. Furthermore, qPCR detected the highest number of malaria infected individuals (n = 293) but none of the infections were detected by LM and/or RDT alone. A total of 145 infections out of 293 positive qPCR (49.5%) results were detected by all three methods (LM, RDT and qPCR) with mean parasite copy numbers of 11,200,000 copies/µL. Only, 18/293 infections (6.1%) detected by two methods (RDT and qPCR only) mean parasite copy numbers of 522,304 copies/µL. The qPCR alone detected 130/193 (44.4%) infections with mean parasite copy numbers of 33,682 copies/µL (Fig. 1). The ability of the PCR to detect the lowest mean parasite density indicates the sensitivity of molecular assays over the routine diagnostic tools.

**Table 3:** Malaria prevalence (both sexual and asexual parasitemia) in symptomatic and asymptomatic children

	Symptomatic (95%)	Asymptomatic (95%)	Difference (95%)	P value
qPCR				
Asexual	89.0 (84.7 to 93.3)	57.5 (50.6 to 64.4)	31.5 (23.4 to 39.6)	<0.001
Sexual	3 (1.6 to 5.4)	14 (11.2 to 16.6)	−11.0 (−16.4 to −5.6)	<0.001
RDT				
Asexual	67.5 (61.0 to 74.0)	14 (9.2 to 18.8)	53.5 (45.4 to 61.6)	<0.001
LM				
Asexual	64.5 (57.9 to 71.1)	8 (4.2 to 11.8)	56.5 (48.9 to 64.1)	<0.001
Sexual	0.5 (−0.5 to 1.5)	2 (0.1 to 3.9)	−1.5 (−3.7 to 0.7)	0.177

P value based on two sample test of proportion

The asymptomatic children had lower risk of infection by asexual stage parasites (OR 0.2, 95% CI 0.1–0.3) and (OR 0.05, 95% CI 0.03–0.08) when tested for qPCR and LM, respectively (Table 4).

**Table 4:** The risk infection between asymptomatic and symptomatic children by the two methods

Group	QPCR	LM
	OR (95% CI)	OR (95% CI)
	Asexual	Asexual
Symptomatic (Ref.)	1.0	1.0
Asymptomatic	0.2 (0.1–0.3)	0.05 (0.03–0.08)

The sensitivity and specificity of RDT was 84.8% (95% CI 68.1–94.9) and 75.8% (95% CI 66.8–83.2) while that of LM was 87.9% (95% CI 71.8–96.6) and 73% (65.8–79.4) for asymptomatic and symptomatic children respectively (Table 5).

**Table 5:** Sensitivity and specificity of LM and RDT (qPCR as a reference method)

Type	RDT		LM	
	Asymptomatic	Symptomatic	Asymptomatic	Symptomatic
Sensitivity	28/33 (84.8%) (95% CI 68.1–94.9)	135/178 (75.8%) (95% CI 66.8–83.2)	29/33 (87.9%) (95% CI 71.8–96.6)	129/178 (72.5%) (95% CI 65.8–79.4)
Specificity	167/167 (100%)	22/22 (100%)	167/167 (100%)	22/22 (100%)

### Gametocyte prevalence by LM and qPCR methods

The detection of *P. falciparum* gametocytes was done by both LM and qPCR methods. Prevalence of gametocytes in asymptomatic school children was 2% by LM while by qPCR was 14% (P < 0.001). Likewise, a sixfold increase on gametocyte prevalence was detected in symptomatic children when the same methods were compared (Table 3).

## Discussion

This study assessed malaria prevalence in school-aged children in Kiwangwa ward in Bagamoyo. Three different malaria diagnostic methods, LM, RDT and qPCR were used to detect *P. falciparum* infections. Using qPCR, the overall malaria prevalence was higher in symptomatic children (89%) compared to asymptomatic ones (57.5%). However, gametocytaemia was high in asymptomatic school children (14%). These findings further support several studies conducted in other malaria endemic countries including; Tanzania [23, 24], Ethiopia [25] and Southern Malawi [26]. These observations clearly point to the necessity of molecular tools in malaria surveillance in order to obtain more reliable estimates of parasite prevalence.

The sensitivity of molecular methods in detecting malaria has been reported in many studies conducted in various malaria settings [24, 27, 28]. These findings indicated further that both RDT and LM are very specific although LM indicated to have a lower detection limit than RDT. Several limitations of LM have been documented [29, 30] such that, its dependency on expertise of microscopy reading, slide preparation methods and its limit of detection something which has been shown by findings from this study. This is an indicator that the quality of malaria case diagnosis needs to be improved since LM is still useful in estimating parasitemia level in blood films as well as detecting non-falciparum infections.

It was within our expectation that school-age children from the health center to have high malaria infections [16, 31]. However, the recorded asymptomatic malaria prevalence of more than 50% among school age children, cannot be ignored since asymptomatic individuals usually harbor submicroscopic parasitaemia [32, 33] which are known to be reservoirs of infections. Although gametocytes do not indicate the intensity of the disease compared to the asexual prevalence, it is one of the indicators of transmission intensity within an area. Even by using the most sensitive diagnostic tool (qPCR), the current study observed, low gametocyte prevalence in symptomatic children (3%) compared to asymptomatic children (14%). Similar observations were also recorded in other studies [5, 34]. Despite the low gametocyte prevalence recorded in the current study, there is evidence that microscopically gametocyte negative samples were able to infect mosquitoes through feeding experiments [35, 36].

The findings from this study indicate the need for conducting a larger or country-wide study to support development and designing more effective elimination programmes in the country. Additionally, the association of mean body weight and malaria infection as it was observed in this study should be considered to further support the explanation of gradual weight loss following prolonged sub-patent illness or infection [37].

The strength of this study lies in the fact that the study assessed malaria prevalence both in asymptomatic and symptomatic school aged children at health facility and schools. Studies conducted in children younger than 10 years in Tanzania focused on either clinical malaria cases [38] or symptomatic individuals [27] and few on asymptomatic individuals [39]. The study recorded both asexual and gametocyte prevalence which measures not only the magnitude of disease but also the potential infective reservoir or transmission intensity in the area, information that is crucial for effective control of malaria in the region. Moreover, this study used three diagnostic methods for parasite detection, which reaffirms the results and confirmed that prevalence of malaria and gametocytes between symptomatic and asymptomatic children varies significantly.

However, there were some limitations of the current study encountered. The recruitment of participants involved only four villages, which were easily accessible what may have restricted the sample size. The study recruited only children aged 6–14 years. The age restriction deprived the study of diversity on age group representation of malaria status both from symptomatic and asymptomatic individuals. Lastly, most of commercially available RDT kits operate by targeting the presence of circulating antigens something which limits differentiation of active infections from resolved ones. Hence, the presence of false positives cannot be completely eliminated.

## Conclusions

Malaria prevalence is high among school aged children, with high malaria cases in symptomatic children. Contrary, gametocytes carriage was high in seemingly healthy children recruited at schools. The use of microscopy for parasite detection usually holds but the use of high sensitive malaria diagnostic tools is important in low malaria settings and where submicroscopic infections are prevalent. The study further established that school-age children are good reservoirs of gametocytes and submicroscopic infections. Hence control efforts should focus on identifying and treating asymptomatic population. Therefore, it is important to identify asymptomatic individuals who are the infective reservoirs in the community in order to achieve malaria elimination in these geographical settings.
